# Comparison of treatment of volar Barton’s fracture with T plate using distal end screws and without distal end screws

**DOI:** 10.12669/pjms.40.8.8799

**Published:** 2024-09

**Authors:** Sajjad Haider, Muhammad Zarak Awais, Eman Salik, Muhammad Tahir Iqbal

**Affiliations:** 1 Sajjad Haider, MS. Department of Orthopedic Surgery, Mayo Hospital, Lahore, Pakistan; 2 Muhammad Zarak Awais, MBBS. Department of Orthopedic Surgery, Mayo Hospital, Lahore, Pakistan; 3 Eman Salik, MBBS. Department of Orthopedic Surgery, Mayo Hospital, Lahore, Pakistan; 4 Muhammad Tahir Iqbal, MBBS. Department of Orthopedic Surgery, Mayo Hospital, Lahore, Pakistan

**Keywords:** Distal end, hand, screws, T-plate, Volar Barton’s fracture

## Abstract

**Objective:**

To compare the functional and radiological outcomes of treatment of volar Barton’s fractures using T plate with and without distal end screws.

**Methods:**

This randomized control trial was conducted at the department of Orthopedics, Mayo Hospital, Lahore, Pakistan from December 2021 to May 2022. Total 60 patients (30 in each group) were randomly allocated to either group A (T-Plate with distal end screws) or B (T-Plate without distal end screws). Patients were followed up on day-0, day-14, day-28 and day-90. Assessments of patients were done by Green O’Brien Score for pain, Modified Warden Score for callus formation on radiographs at 12-weeks of follow-up and Stewart Score for displacement of fracture.

**Results:**

At 90^th^ day, significant difference was seen in Green O’Brien Score (Excellent score: Group-A: 80% vs. Group-B: 90%, p=0.278) while Stewart scores remained comparable (Excellent Score: Group-A: 93.33% vs. Group-B: 96.67%, p=0.221) between treatment groups. Fracture healing assessed with Modified Warden score for score 4 did not show significant difference between both groups at 90^th^ day. i.e. (Group-A: 96.67% vs. Group-B: 96.67%). However, callus formation assessed with Modified Warden score for score 3 showed significant differences between groups at 90^th^ day. i.e. (Group-A: 53.33% vs. Group-B: 86.67%, p=0.001).

**Conclusion:**

Both treatment approaches appear to yield comparable outcomes in terms of clinical assessment, Stewart scores, and fracture healing, with a potential advantage for T-plate without distal end screw in callus formation at the 90th day.

Trial registration: IRCT20221231056999N1.

## INTRODUCTION

Distal radius fractures are quite common, comprising 17.5% of all fracture types.^1^-^3^ Among these fractures, the volar Barton fracture, primarily affecting the distal end of the radius and involving the articular surface, requires precise anatomical reduction.^4^ The name “Barton fracture” originates from John Rhea Barton, an American orthopedic physician from Philadelphia, who bestowed this title upon the fracture in 1838.^5^,^6^ Barton fractures specifically affect the dorsal or volar rim of the distal radius and extend into the intra-articular space.

They are unique in that they involve the articular surface and account for a relatively small portion of distal radius fractures, ranging from 1.2% to 4.2%.^7^,^8^ Additionally, Barton fractures can lead to subluxation or dislocation of the radiocarpal joint.^9^ What sets Barton fractures apart from other distal radius fractures or dislocations is the absence of rupture in the radiocarpal ligaments.^2^ Volar Barton fractures, in particular, are infrequent and are often the result of either high- or low-energy trauma.^5^-^7^ Various treatment regimens are employed both globally and domestically for volar Barton fracture.

International data reveals “open reduction and internal fixation (ORIF)” with Krischner wires, ORIF with a buttress plate or locking plate with screws, ORIF with a volar Ellis plate, arthroscopic internal fixation T-plates, oblique T-plates, and fragment specific plates, as some of the commonly adopted approaches.^4^-^7^ Percutaneous pinning, external fixation, closed reduction and cast immobilization are also suggested.^4^-^7^ In Pakistan, buttress plates, variable angle volar locking plates, T-plates, and T-locking plates are the frequently used treatment approaches.^6^,^10^ This study was performed to compare the functional and radiological outcomes of treatment of volar Barton’s fractures using T plate with and without distal end screws. Both approaches are regularly applied for treating treatment of volar Barton’s fracture so wanted to analyze the outcomes of these two approaches.

## METHODS

This experimental (randomized controlled) trial was carried out at the orthopedic unit of Mayo Hospital, Lahore, Pakistan, from December 2021 to May 2022. The trial was registered at IRCTID: IRCT20221231056999N1,A sample size of 60 patients (30 patients in each group) was calculated using the expected frequency of T-plate with screws as 80%^3^ and T-buttress plate as 44%,^11^ with the level of significance as 5% and the power of the test as 90%. A simple random sampling technique was employed for sample selection by taking a complete history and physical examination of the patients presented in the outpatient department or the emergency department.

### Ethical Approval:

An approval from the “institutional ethical committee” was also obtained (No.843/RC/KEMU, dated: November 14, 2020).

### Inclusion criteria:

Patients of both genders of age between 18-60 years with closed volar Barton’s fracture with Mehara classification types I-II of duration <5 days.

### Exclusion criteria:

Patients with infections, extensive comminution, a compound fracture with significant soft tissue contamination or damage, neurological impairment, serious vascular injuries, ipsilateral upper limb trauma, or patients who refused postoperative physical therapy and follow-up care. Patients having history of severe osteoporosis were also excluded.

All the study patients were briefed about the objective and safety of the study. Written and informed consents were taking assuring them the confidentiality of their data.

Patients were randomly allotted to either Group-A (T-plate with distal end screws) or Group-B (T-plate without distal end screws) by the lottery method. Follow-ups on day 0, 14, 28 and 90 were ensured (through telephonic calls and reminders) once the patients had gone through the intervention. The Modified Henry approach (Trans-FCR) was used to expose the fracture. A skin incision was made over the flexor carpi radialis tendon, incising the tendon sheath. The flexor carpi radialis tendon was exposed, which was retracted ulnar ward and then the far side of the tendon sheath was incised to expose the flexor pollicis longus tendon. The tendon was also retracted in the ulnar ward.

The muscle fibers of the pronator quadratus were incised along their radial sides to expose the fracture. First, the fracture was properly reduced, and then a T-plate of 3.5mm was used. In Group-A patients, all plate screws at the proximal or distal end were used. In Group-B patients, the same plate type was used, only fixing it at the proximal end and leaving the distal end. The closure of the wound was done using a reverse-traction skin-stretching device for the implication of the layered technique. For six weeks, the patient remained immobile with a cast at 20^°^–30^°^ degrees of hyperextension.

“The Green O’Brien score” assessment of pain, range of motion (ROM), grip strength, and activities of the wrist, and was used in this study. “The modified Warden score” was used for radiological assessment. The displacement of the fracture was assessed by Stewart score. The data was analyzed using “Statistical Package for Social Sciences (SPSS)” version 26.0. The study conducted a comparison between two groups using the chi-square test, with a significance threshold set at p≤0.05.

## RESULTS

In a total of 60 cases, 49 (81.7%) were male. The overall mean age was 38.63±9.86 years, ranging 20-58 years. There were no statistically significant differences found with respect to gender or age in between both study groups (p>0.05). Post-surgically, clinical assessment as per Green and O’Brien score revealed no significant difference at 0-day (p=0.056) and at 14^th^ day postoperatively in between both study groups (p=0.135). At 28^th^ (p-value=0.614) and 90^th^ day (p-value=0.278) post-operatively (Table-I).

**Table-I:** Green and O’Brien Score in Treatment Groups.

Follow Ups Interval	Groups	Green and O’Brien Scores				P-Value

		Excellent	Good	Fair	Poor	
0-Day	A	-	4 (13.3%)	12 (40.0%)	14 (46.6%)	0.056
	B		2 (6.7%)	5 (16.7%)	23 (76.7%)	
14^th^ Day	A	-	6 (20.0%)	21 (70.0%)	3 (10.0%)	0.135
	B	-	6 (6.7%)	15 (16.7%)	9 (76.7%)	
28^th^ Day	A	12 (40.0%)	8 (26.7%)	6 (20.0%)	4 (13.3%)	0.614
	B	8 (26.7%)	8 (26.7%)	10 (33.3%)	4 (13.3%)	
90^th^ Day	A	24 (80.0%)	4 (13.3%)	2 (6.7%)	-	0.278
	B	27 (90.0%)	2 (6.7%)	-	1 (3.3%)	

Excellent: 90-100; Good: 80-89; Fair:65-79; Poor: <65.

As per Stewart score, at day-0, (p=0.495) and the 14th day (p-value=0.388), there were no noteworthy distinctions observed between the groups. By the 28th day, 93.3% of patients in Group-A and 96.7% in Group-B exhibited excellent Stewart score (p=0.221), and this trend persisted on the 90th day, with similar results obtained (p=0.221), as depicted in Table-II. We evaluated the stage of fracture healing using the “modified Warden score” which showed no significant differences in both study groups (Table-III).

**Table-II:** Stewart Score in Treatment Groups.

Follow Ups Interval	Groups	Stewart Score				P-Value

		Excellent	Good	Fair	Poor	
0-Day	A	27 (90.0%)	2 (6.7%0	1 (3.3%)	-	0.495
	B	29 (96.7%)	1 (3.3%)	-	-	
14^th^ Day	A	28 (93.3%)	1 (3.3%)	1 (3.3%)	-	0.388
	B	29 (96.7%)	-	-	1 (3.3%)	
28^th^ Day	A	28 (93.3%)	2 (6.7%)	-	-	0.221
	B	29 (96.7%)	-	-	1 (3.3%)	
90^th^ Day	A	28 (93.3%)	2 (6.7%)	-	-	0.221
	B	29 (96.7%)	-	-	1 (3.3%)	

Excellent: 0; Good: 1-3; Fair: 4-6; Poor: 7-12.

**Table-III:** Modified Warden Score (Fracture healing Stage) in Treatment Groups.

Follow Ups Interval	Groups	Modified Warden Score (Fracture Healing Stage)					P-Value

		1	2	3	4	5	
0-Day	A	30 (100%)	-	-	-	-	1
	B	30 (100%)	-	-	-	-	
14^th^ Day	A	30 (100%)	-	-	-	-	1
	B	30 (100%)	-	-	-	-	
28^th^ Day	A	-	-	27 (90.0%)	3 (10.0%)	-	0.687
	B	-	-	26 (86.7%)	4 (13.3%)	-	
90^th^ Day	A	-	-	-	1 (3.3%)	29 (96.7%)	1
	B	-	-	-	1 (3.3%)	29 (96.7%)	

As per “Modified Warden score” on both the zero day and the 14^th^ day, none of the patients exhibited any signs of callus formation. However, by the 28th day, there was a noticeable difference: Group-A had 0% with Score-3, indicating minimal callus, while Group-B had 36.7% with the same score. By the 90th day, Group-A had 53.3% with Score-3, while Group-B had 86.7% (Table-IV).

**Table-IV:** Modified Warden Score (Callus Stage) in Treatment Groups.

Follow Ups Interval	Groups	Modified Warden Score (Callus Stage)					P-Value

		1	2	3	4	5	
0-Day	A	30 (100.0%)	-	-	-	-	1
	B	30 (100.0%)	-	-	-	-	
14^th^ Day	A	30 (100.0%)	-	-	-	-	1
	B	30 (100.0%)	-	-	-	-	
28^th^ Day	A	8 (26.7%)	22 (73.3%)	-	-	-	<0.001
	B	-	19 (63.3%)	11 (36.7%)	-	-	
90^th^ Day	A	-	14 (46.7%)	16 (53.3%)	-	-	0.001
	B	-	2 (6.7%)	26 (86.7%)	2 (6.7%)	-	

## DISCUSSION

Our findings revealed that on 90^th^ day, no significant differences were seen in Green and O, Brien scores (Excellent score: Group-A: 80% vs. Group-B: 90%, p=0.278) and Stewart scores (Excellent Score: Group-A: 93.3% vs. Group-B: 96.7%, p=0.221) between the treatment groups. Fracture healing did not show significant differences between groups at the 90^th^ day, i.e., (Score-5: Group-A: 96.7% vs. Group-B: 96.7%). However, callus formation assessed with the modified Warden score showed significant differences between the groups at the 90^th^ day. i.e. (Score-3: Group-A: 53.3% vs. Group-B: 86.7%, p-value=0.001).

In treating Barton’s fracture of the wrist, AK Aggarwal performed ORIF with a T-plate for the assessment of functional and clinical outcomes. As per their findings, it took 7-10 weeks to heal the fractures. Considering the functional criteria nine cases resulted in excellent, five good, and two fair.^11^ The volar Barton’s fracture fixation was assessed in a study for functional and radiological outcomes using a variable locking plate, and it was found that excellent and good outcomes were found in 65% and 30% cases while 5% reported fair outcomes.^12^

In two clinical reports, rapid healing of the fracture and a low incidence of bone grafting were observed using volar fixed angle plating, making tendon complications less common.^13^,^14^ In a study, they compared the volar fixed angle T-plate with the contralateral side in unstable distal radius fractures and noted that, at the 10 months follow-up, it had maintained flexion-extension in 81%, radial-ulnar deviation in 84%, pronation-supination in 91%, and grip strength in 74%.^15^ The above-mentioned studies have reported excellent outcomes with T-plate. However, none of the studies have compared the use of a T-plate with and without distal end screw fixation, which makes this study the first of its kind in which this concept was investigated.

Another local study reported that, up until recently, treating unstable distal radius fractures without angle-stable screws has been successful with the Palmer T-plate.^16^ In an Indian, using “Modified Clinical Scoring System of Green and O’Brien”, 84.4% positive results were achieved while the functional assessment showed that 75% of the participants did not have any issues.^17^ Numerous studies have been done to treat unstable distal radius fractures, and each one has produced excellent results, however, the best course of treatment for such unstable fractures is still up for debate.^18^,^19^ Dorsal plates are used to treat unstable distal radius fractures; however, they often necessitate spongioplasty, fragment exposure, and eventual implant removal.

However, using the T-plate and the palmar technique, articular surface rebuilding and restoration can be accomplished with ease. Keeping in mind the above discussion, good anatomical reduction seems to be achievable using a T-plate, but it needs good care to evade the complications relating to tendon rupture.

### Limitations:

It includes a single-center study with a relatively small sample size. The follow-up period of 90 days was also relatively short, so further prospective trials involving long-term follow-ups will further add to the findings of this study.

## CONCLUSION

Both treatment approaches appear to yield comparable outcomes in terms of clinical assessment, Stewart scores, and fracture healing, with a potential advantage for T-plate without distal end screw in callus formation at the 90th day. Further research with larger sample sizes may provide more insights.

### Authors Contribution:

**SH:** Data Collection, responsible for data’s integrity.

**MZA:** Data collection, drafting the manuscript.

**ES & MTI:** Data collection, literature review.

All authors have read and approved the final version to be published.
